# Local T/B cooperation in inflamed tissues is supported by T follicular helper-like cells

**DOI:** 10.1038/ncomms10875

**Published:** 2016-02-26

**Authors:** Dana Vu Van, Katja C. Beier, Lea-Jean Pietzke, Maysun S. Al Baz, Randi K. Feist, Stephanie Gurka, Eckard Hamelmann, Richard A. Kroczek, Andreas Hutloff

**Affiliations:** 1 Chronic Immune Reactions, German Rheumatism Research Centre, A Leibniz Institute, Charitéplatz 1, 10117 Berlin, Germany; 2 Molecular Immunology, Robert Koch Institute, Nordufer 20, 13353 Berlin, Germany; 3 Pediatric Pneumology and Immunology, Charité University Medicine, Augustenburger Platz 1, 13353 Berlin, Germany; 4 Children's Hospital of Ruhr University, Alexandrinenstraße 5, 44791 Bochum, Germany

## Abstract

Autoimmune diseases and other inflammatory conditions are characterized by large lymphocytic tissue infiltrates in which T and B cells can be found in close contact. Here, using a murine airway inflammation model, we compare antigen-specific T and B cells in lung tissue versus lung-draining lymph node. In the lung we identify a B-cell population exhibiting a classical germinal centre phenotype without being organized into ectopic lymphoid tissue. By contrast, classical CXCR5^+^ Bcl-6^+^ T follicular helper cells are not present. Nevertheless, lung-infiltrating T cells exhibit follicular helper-like properties including the potential to provide help to naive B cells. The lung tissue is also a survival niche for memory T and B cells remaining in residual peribronchial infiltrates after resolution of inflammation. Collectively, this study shows the importance of T/B cooperation not only in lymph nodes but also in inflamed peripheral tissues for local antibody responses to infection and autoimmunity.

## Abstract (web summary)

In secondary lymphoid organs T follicular helper (Tfh) cells help B cells to develop into memory B and plasma cells. Here, the authors show that inflamed lung becomes a reservoir of activated B cells with a germinal centre phenotype, and T cells that exhibit Tfh-like properties despite not expressing classical Tfh markers.

## Graphical abstract

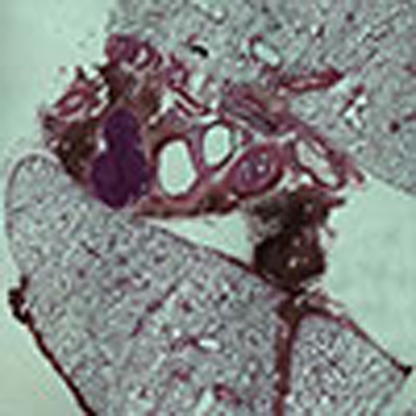

Leukocytic infiltrates in peripheral tissues are frequently found in autoimmune conditions like rheumatoid arthritis, systemic lupus erythematosus, Sjögren syndrome and multiple sclerosis, but also in the lungs of asthma patients. These infiltrates typically contain antigen-specific T and B cells, as well as different cell populations of the innate immune system, and contribute substantially to tissue destruction and immunopathology. B cells produce (auto-) antibodies locally and also seem to play a role as antigen-presenting cells for T lymphocytes in the periphery. T cells in turn produce proinflammatory cytokines, which attract tissue-destructive granulocytes and also affect non-lymphoid cells, for example, goblet cells to produce mucus in the case of airway inflammation. However, an often neglected function of T cells in inflamed tissues is their potential to provide help to antigen-specific B cells.

This helper function of T cells is the major role of T follicular helper (TFH) cells, a specialized population of T cells in secondary lymphoid organs (SLOs)^1^. This T-cell population is crucial for B-cell maturation and differentiation in the germinal centre (GC) response^2^. Without TFH cells, no affinity-matured long-lived plasma cells and memory B cells are generated. These two terminally differentiated B-cell populations are the basis for protective immunity; however, they can pose a major problem when producing autoreactive antibodies. Therefore, TFH cells are an attractive target for the treatment of autoimmune and other inflammatory diseases.

Under certain conditions, SLO-like structures can develop in inflamed tissues. They are known as ‘ectopic lymphoid tissue' or as ‘induced bronchus-associated lymphoid tissue' in the lungs^3^,^4^. Ectopic lymphoid tissues represent highly ordered structures with separate T- and B-cell zones. Another important characteristic is the presence of follicular dendritic cells (FDCs) similar to follicles in SLO. Ectopic lymphoid tissues exhibit many features of SLO, including formation of germinal centres in which T and B cells cooperate^5^.

However, the development of ectopic lymphoid tissue in inflamed tissues is an exceptional case, which requires experimental settings with strong stimuli or other facilitators like a viral infection^3^. In human autoimmune conditions, fully differentiated ectopic follicles are only rarely observed^6^,^7^. Nevertheless, FDC-negative lymphocytic infiltrates contain T and B cells in very close contact raising the question whether T/B cooperation can also take place in infiltrates not exhibiting the features of ectopic lymphoid tissue.

To analyse the cooperation of antigen-specific T and B cells in inflamed tissues in more detail, we use a novel lung inflammation mouse model, which makes it possible to analyse and compare the interaction of antigen-specific T and B cell simultaneously in inflamed lung tissue, as well as in the lung-draining lymph nodes. With this model we identify the inflamed lung tissue as the major reservoir of antigen-specific T and B cells. The lung tissue does not only contain antigen-specific plasma cells but also a population of GC-like B cells. In contrast, no classical CXCR5^+^ Bcl-6^+^ TFH cells are present in the lung. However, we identify a population of lung-infiltrating helper T cells, which seem to take over the functions of TFH cells. Finally, we show that the lung tissue is an important survival niche for antigen-specific memory T and B cells, which might be important for fast local secondary responses.

## Results

### Antigen-specific T and B cells accumulate in lung tissue

**Figure 1:**
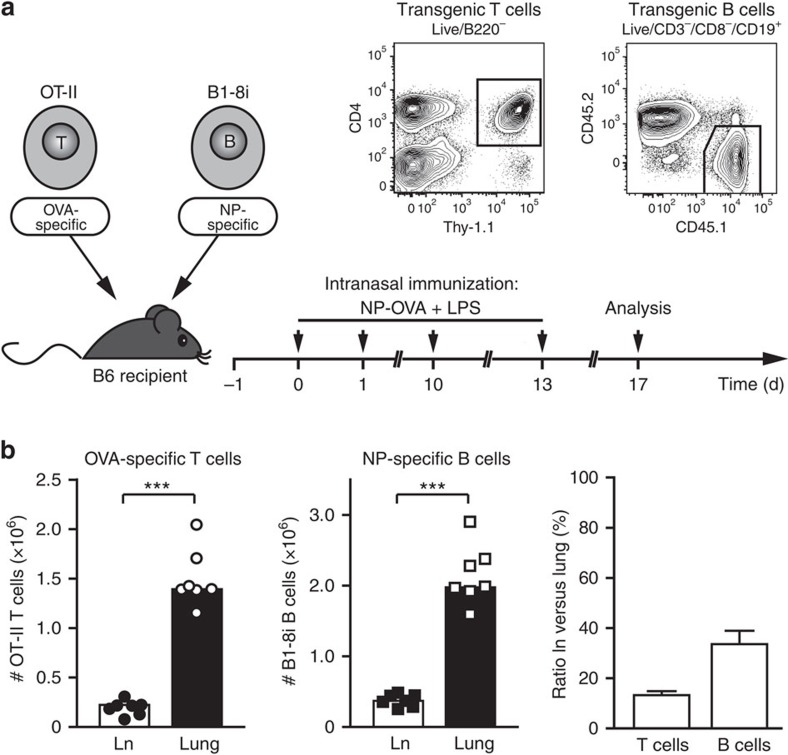
Inflamed lung tissue is the major reservoir of antigen-specific T and B cells. (**a**) General set-up for the airway inflammation model. Preactivated OT-II T cells and naive B1-8i/kappa light chain knockout B cells were transferred into syngeneic C57BL/6 mice. OT-II cells express a T-cell receptor specific for OVA, B1-8i cells have a knock-in for the germline VH186 heavy chain, which recognizes the hapten NP in combination with any lambda 1 light chain^31^. On the indicated days, recipient mice were immunized i.n. with an NP–OVA conjugate and LPS. Cells from lungs and lung-draining lymph nodes were typically analysed on day 17 when airway inflammation was fully established. Antigen-specific T and B cells were identified by flow cytometry using the congenic markers Thy-1.1 and CD45.1, respectively. (**b**) Total numbers of antigen-specific T and B cells in lung-draining lymph node (Ln) and lung tissue on day 17. Shown are absolute cell numbers of a representative experiment and the ratio of antigen-specific T and B cells in lymph node versus lung (pooled data from 6 experiments with 34 animals). Error bars show s.e.m. ***P<0.001 by Mann–Witney *U*-test.

The low natural frequency of antigen-specific T and B cells makes it difficult to analyse their interaction in an inflammatory reaction. Therefore, we developed an *in vivo* T/B cooperation system, in which ovalbumin (OVA)-specific T cells were co-transferred with nitrophenol (NP)-specific B cells into immunocompetent recipient mice (Fig. 1a). After adoptive transfer, recipient mice were challenged intranasally (i.n.) with an NP–OVA conjugate as antigen and lipopolysaccharide (LPS) as an adjuvant. This system makes it possible to analyse antigen-specific T and B cells simultaneously in lung tissue and lung-draining lymph nodes, using the congenic markers Thy-1.1 and CD45.1 (in all subsequent figures, antigen-specific refers to cells gated on either CD4^+^ Lin^−^ Thy-1.1^+^ or CD19^+^ Lin^−^ CD45.2^−^ CD45.1^+^). One to two days after antigen challenge, antigen-specific T and B cells accumulated in the lung-draining lymph nodes where they proliferated vigorously. Although cells evenly distributed to all SLOs on transfer, no activated antigen-specific T and B cells were found in non-draining organs like the spleen. In the lungs, activated antigen-specific T and B cells appeared around day 5 (Supplementary Fig. 1). After repeated antigen challenges, a stable population of lung-infiltrating T and B cells developed, which did not change much over time. Therefore, we routinely analysed recipient mice on day 17 for antigen-specific T and B cells in the draining lymph nodes versus the inflamed lung. At that time, more than 80% of all antigen-specific T and 60% of B cells were found in the lungs, identifying the inflamed tissue as the major reservoir of antigen-specific T and B cells (Fig. 1b).

### Infiltrates harbour T and B cells in intimate contact

**Figure 2:**
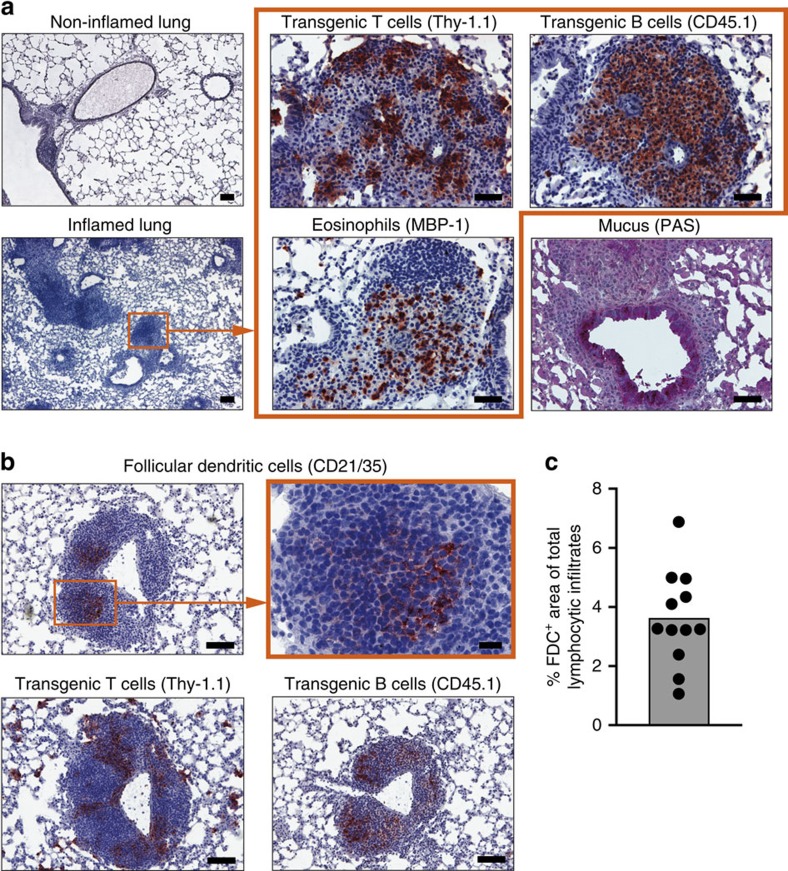
Lung infiltrates contain antigen-specific T and B cells in close contact. (**a**) Cryosections from non-inflamed and inflamed lung tissues (day 17) were stained with haematoxylin to visualize cellular infiltrates. The images on the right show serial sections of the highlighted perivascular infiltrate or the nearby bronchus in higher magnification. Immunostaining (red) for OVA-specific T cells, NP-specific B cells and eosinophils was performed using the indicated markers. Production of mucus was visualized by periodic acid–Schiff staining. Scales bars, 200 μm for the low-resolution images (original magnification × 5) and 50 μm for the high-resolution images (original magnification × 20). (**b**) Ectopic lymphoid tissue was identified by staining of serial cryosections for FDC and antigen-specific T and B cells using the indicated markers. Scale bars, 100 μm for the low-resolution images and 20 μm for the high-resolution image (original magnification × 20). Images are representative of 2–4 independent experiments with 10–18 animals total. (**c**) Area of FDC-containing regions as percentage of total infiltrate area, quantified as shown in Supplementary Fig. 2. Each dot represents data from a single pulmonary lobe.

After repeated i.n. challenge with antigen, we observed the typical signs of chronic lung inflammation, such as appearance of large lymphocytic infiltrates and airway narrowing with increased mucus production (Fig. 2a). Immunohistological examination on day 17 revealed large lymphocytic clusters containing eosinophils and antigen-specific T cells located adjacent to antigen-specific B cells. Under chronic inflammatory conditions, lymphocytic infiltrates can develop further into ectopic lymphoid tissue, characterized by the presence of FDC^3^,^4^. Therefore, we stained the lung infiltrates for CD21/35 (complement receptor 2), which is expressed on FDC at high density. More than 96% of infiltrates were negative for CD21/35 (Fig. 2c and Supplementary Fig. 2). Only some very large infiltrates located near the central bronchus stained positive for FDC markers (Fig. 2b and Supplementary Fig. 2). In contrast to the FDC-negative infiltrates composed of loose aggregates of T and B cells, these CD21/35-positive infiltrates showed highly organized separate T- and B-cell zones (Fig. 2b, lower panel). It is well established that T/B cooperation can take place in these SLO-like structures^3^,^4^. However, the close contact of antigen-specific T and B cells in the predominating FDC-negative loose aggregates raised the question whether local T/B cooperation might take place there as well.

### Lung tissue contains B cells with a GC-like phenotype

**Figure 3:**
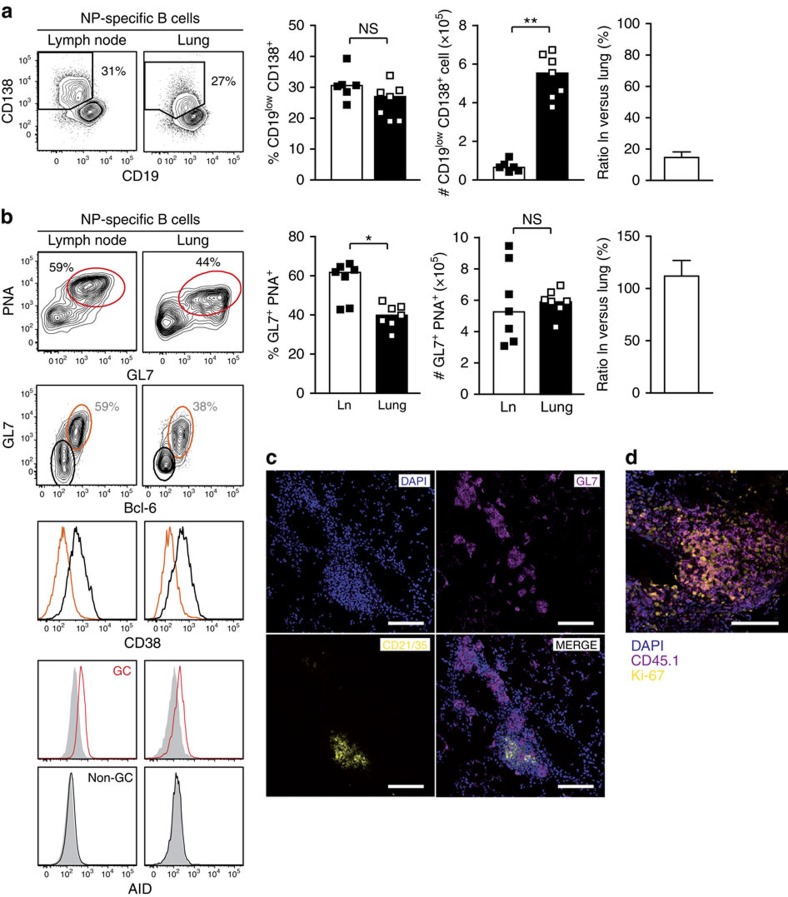
Similar phenotype of B cells in lymph node and lung tissue. Flow cytometric analysis (day 17) of antigen-specific (CD45.1^+^) B cells from lung-draining lymph nodes and lung tissue. (**a**) Comparison of plasma cells, defined as CD19^low^ CD138^+^, from lymph nodes and lungs. Shown are flow cytometric plots, and the frequency and absolute numbers of NP-specific B cells with a plasma cell phenotype from a representative experiment and the ratio of plasma cells in lung-draining lymph nodes as pooled data from 3 experiments with 20 animals total. (**b**) Comparison of GC/GC-like B cells from lymph nodes (Ln) and lung tissue. Frequency and absolute numbers from a representative experiment, and relative distribution of GL7^+^ PNA^+^ B cells between lymph node and lung as pooled data from 4 experiments with 26 animals. For further characterization, cells were co-stained for CD38 and intracellular Bcl-6 or activation-induced cytidine deaminase (AID). CD38 expression is shown as histograms on GL7/Bcl-6 double-negative (black) versus double-positive (orange) cells. AID expression is shown on GL7/PNA-positive (red line) and -negative (black line) cells relative to an isotype control (filled grey histogram; concatenated flow cytometry data from five animals). (**c**) Staining of lung tissue containing ectopic lymphoid tissue defined by the presence of FDC (CD21/35^+^, yellow) and a greater number of unstructured infiltrates. Staining with the GC B-cell marker GL7 is shown in magenta. (**d**) Co-staining of transgenic B cells (magenta) and the cell proliferation marker Ki-67 (yellow) in an FDC-negative infiltrate. Scale bars, 100 μm, original magnification × 20. Data are representative for at least two independent experiments. Error bars show s.e.m. NS, not significant: *P*≥0.05, *P<0.05, **P<0.01 by Mann–Witney *U*-test.

To find indications for T/B cooperation in the inflamed tissue, we compared the phenotype of lung-infiltrating B cells with that of lymph node B cells. First, we looked for antibody-secreting cells, which are generated both in the extrafollicular and GC B-cell responses. On day 17, up to 30% of antigen-specific B cells in the draining lymph nodes expressed CD138 and had downregulated CD19, a characteristic feature of plasma cells (Fig. 3a). The lung tissue contained a very similar fraction of antigen-specific plasma cells. However, when considering the higher absolute numbers of antigen-specific B cells (compare Fig. 1b), the lung tissue turned out to contain eight times more plasma cells than the lung-draining lymph node. Intracellular staining of immunoglobulin subclasses revealed that antigen-specific plasma cells from both sites were mainly switched towards IgG1 and IgA, with few cells producing IgG2a and IgE (Supplementary Fig. 3). Histological analysis showed that CD138-positive plasma cells were equally found in FDC-positive and -negative infiltrates (Supplementary Fig. 4).

Next, we analysed the generation of GC B cells. Around 60% of the transgenic B cells in the lymph node displayed a GC phenotype, defined by staining with peanut agglutinin (PNA) and the monoclonal antibody GL7 (Fig. 3b). Looking for these classical GC B-cell markers in the lungs, we surprisingly found a prominent population of PNA/GL7 double-positive cells, which only differed by a slightly reduced binding of PNA (Fig. 3b). Although their frequency in the lung was lower, their absolute number was similar to that in the lymph node. GC B cells have been detected in ectopic lymphoid structures of inflamed tissues^5^. However, the high percentage of PNA^+^/GL7^+^ cells in our inflammation model was unexpected given the fact that the vast majority of lung infiltrates consisted of loose T/B aggregates and not ectopic lymphoid tissue. Therefore, we analysed additional markers that are more specific for GC B cells. The GL7^+^ B cells in the lung expressed the transcription factor Bcl-6, which is a key regulator of GC B-cell differentiation^2^, and had downregulated CD38, another attribute of GC B cells^8^. Moreover, PNA/GL7 double-positive cells in lymph node and lung expressed the activation-induced cytidine deaminase, which is involved in somatic hypermutation of GC B cells (Fig. 3b).

To define the localization of the GC-like B cells, we analysed a lung section near the central bronchus, which contained both ectopic lymphoid tissue and unstructured infiltrates (Fig. 3c). As expected, some GL7^+^ GC B cells were present in ectopic lymphoid tissue containing CD21/35^+^ FDC. However, most GL7^+^ B cells were found in infiltrates without FDC, demonstrating that GC-like B cells can exist outside GC structures (Fig. 3c). This explains how up to 40% of lung-infiltrating B cells displayed a GC phenotype although 96% of all lung infiltrates in our model were composed of loose T/B aggregates. Staining for the cell cycle marker Ki-67 revealed that B cells within these infiltrates were highly proliferating (Fig. 3d). This further indicates that active T/B cooperation takes place within these loose lung infiltrates.

Finally, we sequenced the transgenic B-cell receptor heavy chain from FDC-positive and -negative infiltrates obtained by laser microdissection. Both types of infiltrates contained hypermutated B cells. The ratio of replacement versus silent mutations (1.24 versus 1.33) and also the percentage of clones with the affinity-determining tryptophan to leucine mutation at codon 33 (60% versus 52%) were similar in FDC-positive and -negative infiltrates.

Taken together, our data show that B cells in lymph nodes and lung are similar in their phenotype. However, in contrast to lymph nodes, where PNA^+^/GL7^+^ cells are present only in GC, we identified GC-like B cells outside of ectopic lymphoid structures in inflamed lung tissue.

### Lung-infiltrating T cells are no classical TFH cells

**Figure 4:**
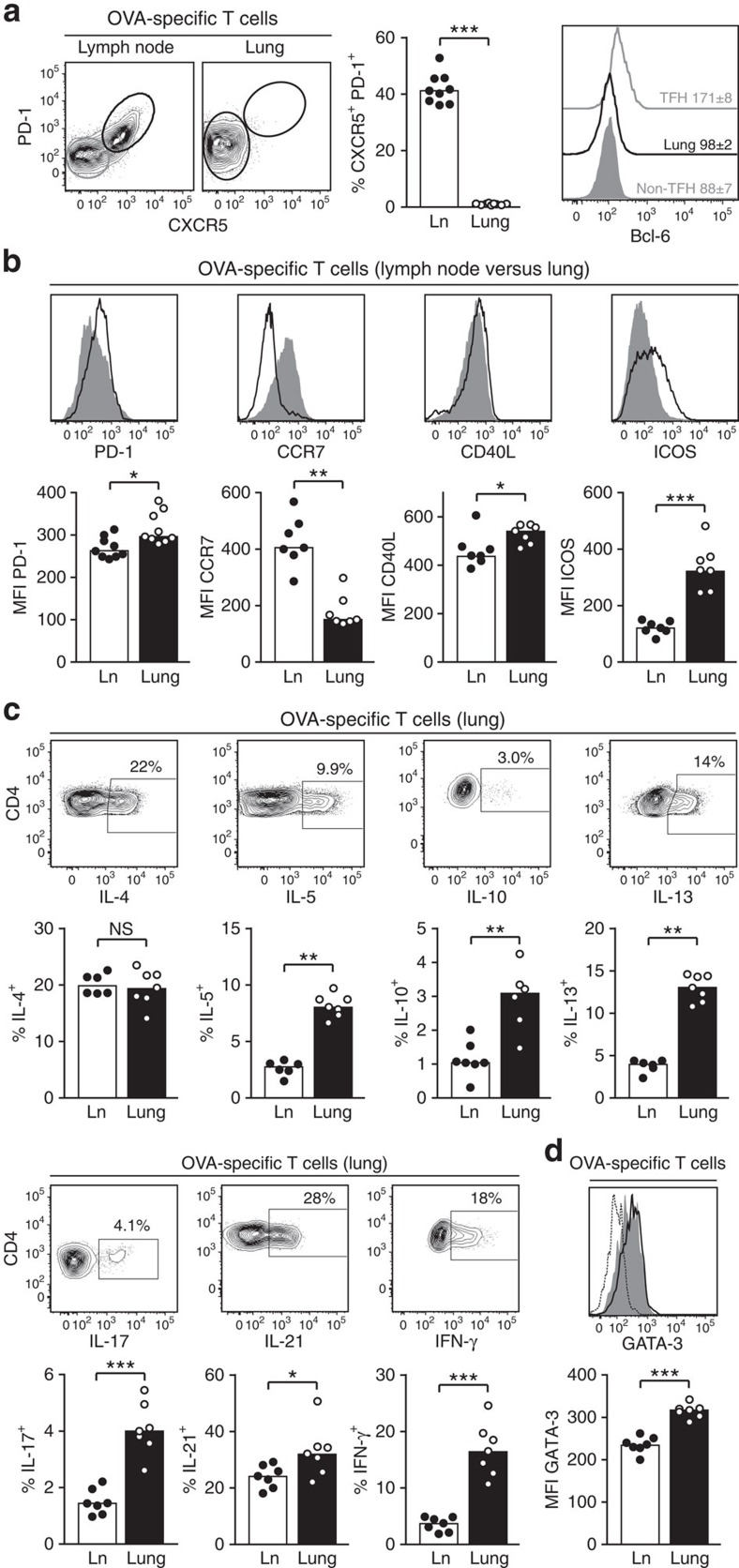
Lung tissue does not contain classical TFH cells. Comparison of antigen-specific (Thy-1.1^+^) T cells from lymph nodes (Ln) and lung tissue. (**a**) Analysis of CXCR5/PD-1 expression. The right panel shows intracellular staining for Bcl-6 in lymph node TFH cells (CXCR5^+^/PD-1^+^) and non-TFH cells (CXCR5^−^/PD-1^−^), and in the whole population of lung-infiltrating T cells. The inset numbers indicate the mean fluorescence intensity (MFI)± s.e.m. (**b**) Expression of PD-1, CCR7, CD40L (intracellular staining, no restimulation) and ICOS on lymph node (grey-filled histograms) versus lung-infiltrating (black open histograms) T cells. (**c**) Intracellular staining for IL-4, IL-5, IL-10, IL-13, IL-21 and IFN-γ after short-term restimulation with OVA peptide *in vitro*. A representative staining from lung-infiltrating T cells is shown for all cytokines. (**d**) Intracellular staining for GATA-3 comparing the MFI between lymph node and lung T cells (dotted line=GATA-3 staining on non-antigen-specific (endogenous) lymph node CD4^+^ T cells). Data are representative of 3–4 independent experiments with 7–9 animals per experiment. NS, not significant: *P*≥0.05, *P<0.05, **P<0.01, ***P<0.001 by Mann–Witney *U*-test.

As the generation of GC B cells in lymph nodes critically depends on the presence of TFH cells, we analysed the proportion of transgenic T cells expressing the TFH markers CXCR5 and PD-1. In the lung-draining lymph nodes about 40% of T cells displayed a TFH phenotype (Fig. 4a). In contrast, this population was missing completely in the inflamed lung tissue. Interestingly, only CXCR5 was absent on lung T cells, whereas PD-1 was equally expressed on lung as well as on lymph node T cells (Fig. 4b). To define TFH cells more precisely we stained their master transcription factor Bcl-6. As expected, antigen-specific CXCR5^−^ PD-1^−^ T cells in the lymph node were negative for Bcl-6, whereas a significant Bcl-6 expression was found in the CXCR5^+^ PD-1^+^ subset. In contrast, lung-infiltrating T cells did not express Bcl-6 at all (Fig. 4a, right panel).

To further compare lung-infiltrating and lymph node T cells, we analysed additional markers that are typically expressed by TFH cells: the chemokine receptor CCR7, which is also involved in the positioning of TFH cells in the B-cell zones of SLO, and CD40L and ICOS, which are important effector molecules. Interestingly, lung-infiltrating T cells had downregulated CCR7 to an even stronger extent than antigen-specific T cells in the lymph node (Fig. 4b). At the same time, expression levels of CD40L and ICOS were higher on lung-infiltrating T cells compared with lymph node T cells.

T-cell help to B cells is mediated by CD40L and secretion of interleukin (IL)-4 and IL-21. To assess the cytokine-producing potential of lung-infiltrating and lymph node T cells, these cell populations were re-stimulated with antigen *in vitro*. IL-4 and IL-21 were the two dominating cytokines, produced by more than 20% of antigen-specific T cells (Fig. 4c). While IL-4 was produced to a similar amount by lymph node and lung T cells, IL-21 production by lung-infiltrating T cells was in all experiments significantly higher than by antigen-specific T cells isolated from the draining lymph nodes. Interestingly, expression of additional effector cytokines such as IL-5, IL-10, IL-13, IL-17 and interferon (IFN)-γ was largely confined to lung T cells with an at least threefold higher expression than antigen-specific T cells from the draining lymph nodes. The higher expression of Th2 effector cytokines by lung T cells was associated with a significantly increased expression of the master transcription factor GATA-3 (Fig. 4d).

In contrast to the classical TFH cells in the lymph node, there was no cell surface marker on lung-infiltrating T cells identifying a subset with particular B helper capacity. Therefore, we analysed co-expression of different cytokines. IL-21 turned out to be a lead cytokine for lung effector T cells. IL-4-, IL-10-, IL-13- and IFN-γ-producing cells were highly enriched in the IL-21-positive compared with the IL-21-negative subset (Supplementary Fig. 5). As a second cytokine, the IL-21/IL-4 double-positive versus the IL-21^+^ IL-4^−^ subset demarcated cells with highest expression of IL-10 and IL-13.

To demonstrate that the B helper potential of lung-infiltrating T cells was not a consequence of the preactivation of OT-II T cells under Th2-polarizing conditions, we ran our airway inflammation model also with the transfer of naive T cells. Under these conditions, all previous observations could be reproduced (Supplementary Fig. 6). We found a GC-like B-cell population in the lung but no CXCR5^+^ TFH cells. The cytokine profile partly changed with almost no IL-4- and IL-13-producing cells. Instead, a population of up to 30% IL-17-producing cells was generated in the lung, which is a typical reaction for antigens taken up via an intranasal route^9^. Importantly, production of IL-21 was unchanged with around 30% IL-21^+^ cells. Again, IL-21 turned out to be a lead cytokine for lung T cells, with higher IL-17 and IFN-γ production in the IL-21-positive versus -negative subset.

Collectively, in striking contrast to the presence of GC-like B cells in the lung, no classical CXCR5^+^ Bcl-6^+^ TFH cells could be found. However, regarding other markers, lung-infiltrating T cells shared many similarities with TFH cells and had a high B helper potential by expressing CD40L, IL-4 and especially IL-21. Lung-infiltrating T cells produced additional effector cytokines like IL-5, which functions not only as a chemoattractant for eosinophils but is also important for the class-switch of B cells towards IgA^10^.

### Lung-infiltrating T cells provide help for B cells

**Figure 5:**
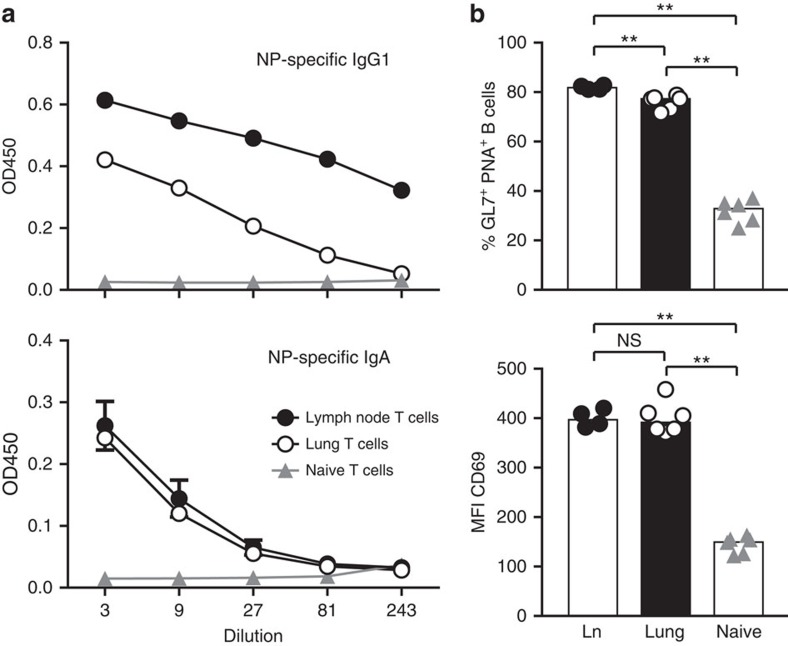
Lung-infiltrating T cells provide B-cell help. Sorted antigen-specific (Thy-1.1^+^) effector T cells (day 17) from lungs or lymph nodes (Ln), or naive OT-II cells were co-cultured with naive NP-specific B cells in the presence of NP–OVA for 7 days. Thereafter, (**a**) NP-specific IgG1 and IgA were measured by ELISA and (**b**) B cells were stained with PNA, GL7 or anti-CD69, and analysed by flow cytometry. Shown are representative results from three independent experiments with six replicates each. Error bars show s.e.m. NS, not significant: *P*≥0.05, **P<0.01 by Mann–Witney *U*-test.

To functionally test their capacity for B-cell help, antigen-specific T cells from lung-draining lymph nodes or lung tissue were sorted and co-cultured with naive NP-specific B cells in the presence of cognate antigen. After 7 days, B cells co-cultured with lung T cells had produced large amounts of IgG1, although not quite to the extent as B cells co-cultured with T cells from lymph nodes (Fig. 5a, upper panel). However, production of IgA was similarly high in both groups (Fig. 5a, lower panel). Moreover, lung T cells induced the ligands for GL7 and PNA, and the activation marker CD69 on the co-cultured B cells to the same extent as lymph node T cells (Fig. 5b). Taken together, lung-infiltrating T cells are fully capable to provide B-cell help, although they lack the prototypic TFH cell markers CXCR5 and Bcl-6.

### The lung is a major reservoir for memory B cells

**Figure 6:**
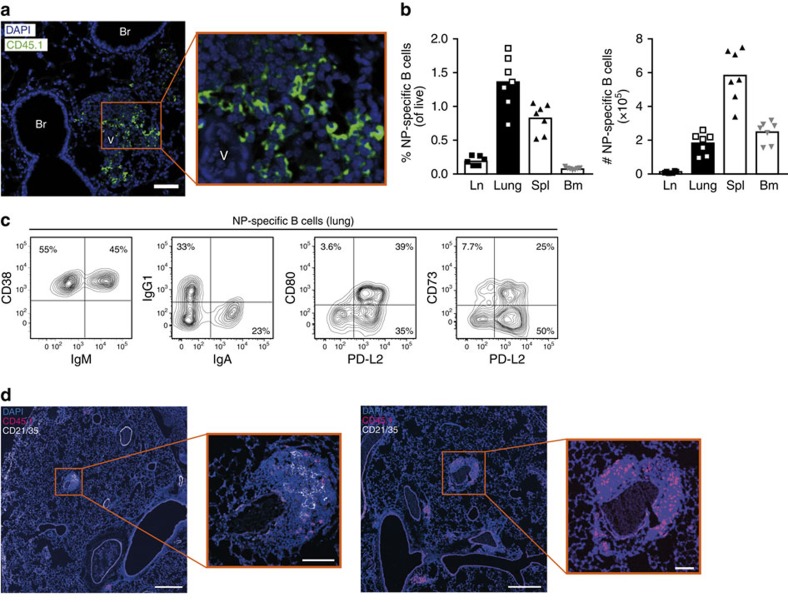
Lung tissue is a major reservoir for antigen-specific memory B cells. Analysis of lung-resident B cells 28 days after the last antigen challenge (day 41). (**a**) Histological analysis of lung tissue showing a peribronchial infiltrate. Antigen-specific B cells (CD45.1^+^, green) were preferentially found in the proximity of vessels. Br, bronchus, V, vessels. Scale bar, 50 μm, original magnification was × 20. (**b**) Frequencies and absolute numbers of antigen-specific (CD45.1^+^) B cells isolated from lymph node (Ln), lung, spleen (Spl) and bone marrow (Bm) on day 41. (**c**) Characterization of lung-resident antigen-specific B cells showing representative staining for CD38, IgM, IgG1, IgA and the memory markers PD-L2, CD80 and CD73. Representative data from two independent experiments with six mice per experiment are shown. (**d**) Co-staining of antigen-specific B cells (CD45.1) and FDC (CD21/35) in lung tissue on day 42. Scale bars, 500 μm in the overview and 100 μm in the detail enlargement.

After looking at T/B cooperation during the effector phase of airway inflammation, we analysed antigen-specific T and B cells during the memory phase. Even as late as day 41, 4 weeks after last antigen application, perivascular lymphocytic infiltrates could still be found in the lung (Fig. 6a). These infiltrates contained substantial numbers of antigen-specific B cells. At the same time, barely any NP-specific B cells were detectable in the lymph node (Fig. 6b). In contrast to the effector phase, where activated antigen-specific cells were exclusively found in the lung and lung-draining lymph node, memory B cells were now also present in spleen and bone marrow. While the highest frequency of memory B cells was found in the lung, the majority of memory B cells in absolute numbers resided in the spleen taking into account the higher cellularity of this organ (Fig. 6b).

We analysed additional markers to characterize these lung-resident antigen-specific B cells. While during the effector phase almost all antigen-specific B cells in the lungs displayed either a GC or plasma cell phenotype, CD138^+^, intracellular Ig^+^ or GL7^+^, PNA^+^ and CD38^low^ cells were no longer present at this late time (Fig. 6c and Supplementary Fig. 7). Half of the antigen-specific B cells in the lungs expressed surface IgM, whereas the others were class-switched towards either IgG1 or IgA (Fig. 6c). Around 40% of all B cells expressed CD80, which is regarded as a marker for highly mutated memory B cells^11^. In addition, we analysed the expression of PD-L2 and CD73, which are also expressed by subsets of memory B cells^12^. Altogether, more than 85% of the B cells expressed PD-L2, CD73 or CD80, and can therefore be regarded as typical memory B cells, identifying the lung as an important niche for antigen-specific memory B cells.

Since <10% of the antigen-specific B cell numbers found during the effector phase remained at day 41, we had to take into account the possibility that they might be exclusively located in the FDC-positive infiltrates near the central bronchus. However, like in the effector phase, the vast majority of infiltrates were FDC-negative. Co-staining of CD45.1 and CD21/35 revealed that also in the memory phase the great majority of antigen-specific B cells resided in FDC-negative infiltrates (Fig. 6d).

### Memory T and B cells are in close contact in the lung

**Figure 7:**
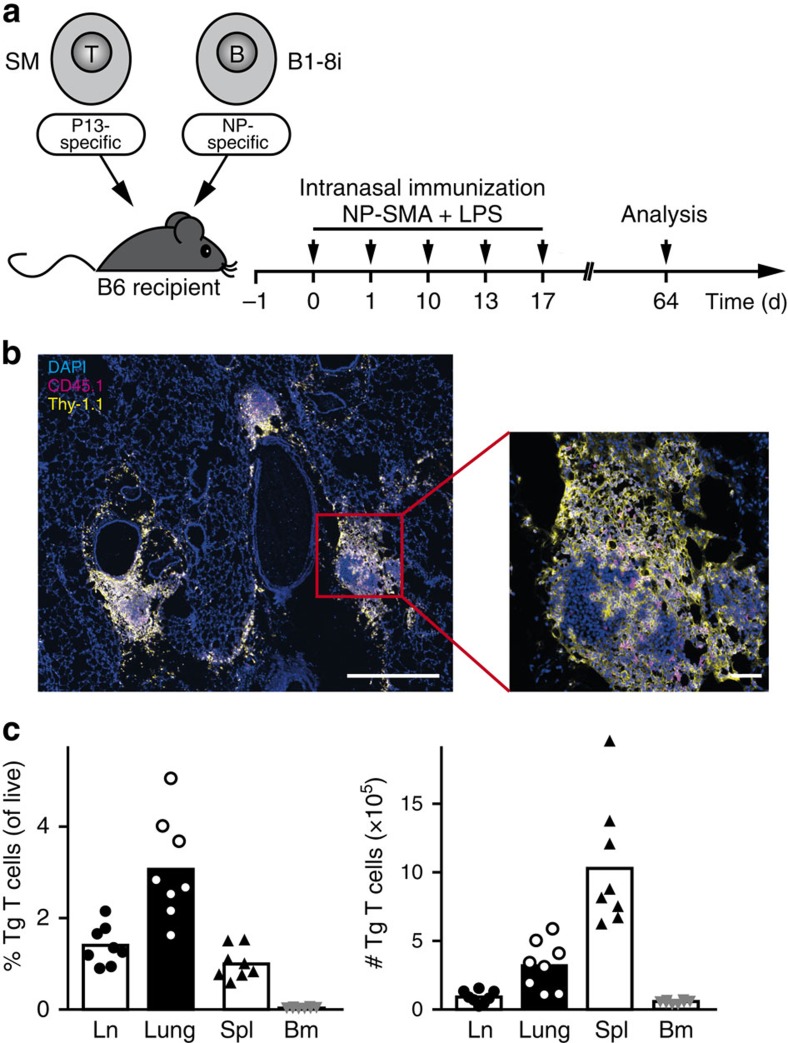
Clusters of antigen-specific memory T and B cells remain in the non-inflamed lung. (**a**) Smarta T-cell receptor transgenic (Tg) T cells were co-transferred with B1-8i B cells into C57BL/6 recipients. Mice were repeatedly challenged i.n. with NP and Smarta peptide coupled to mouse serum albumin as non-immunogenic carrier and analysed 47 days (d) after the last challenge. (**b**) Histological analysis of lung tissue showing antigen-specific T cells (Thy-1.1^+^) in yellow and B cells (CD45.1^+^) in magenta. Scale bars, 500 μm in the overview and 50 μm in the detail enlargement. (**c**) Frequencies and absolute numbers of antigen-specific T cells isolated from lymph node (Ln), lung, spleen (Spl) and bone marrow (Bm). Representative data from 2 independent experiments with 14 mice total.

To additionally analyse antigen-specific memory T cells, we switched our system to the adoptive transfer of Smarta T-cell receptor-transgenic instead of OT-II T cells, which allows for long-term tracking in vivo^13^ (Fig. 7a). We co-stained lung sections for antigen-specific T and B cells on day 64 (47 days after the last antigen challenge). Like in the effector phase (compare Fig. 2a) both cell types were still found in close contact within the residual infiltrates (Fig. 7b). Antigen-specific memory T cells were recovered not only from lung tissue but also from lung-draining lymph node, spleen and bone marrow (Fig. 7c). Consistent with the distribution of the memory B cells, the highest frequency of memory T cells was found in the lung, however, the highest absolute number was in the spleen.

## Discussion

The interaction of T and B cells in SLO as well as in ectopic lymphoid tissue, which are structurally and functionally similar, has been investigated in great detail in the past. However, it is still unclear whether T/B cooperation can also take place in unordered T/B infiltrates lacking the characteristics of SLO, such as clearly separated T- and B-cell zones and the presence of FDC. This question is of major interest, because these unstructured T/B aggregates represent the vast majority of infiltrates in inflamed tissues in autoimmune and allergic diseases. While early studies reported the presence of FDC-positive ectopic lymphoid tissue in around 25% of all synovial biopsies from rheumatoid arthritis patients^14^, a more recent study found fully differentiated follicles in only 6% of the biopsies^6^. The predominant type of infiltrates was characterized as either diffuse T- and B-cell infiltrates or T/B aggregates lacking FDC structures. Similar observations were made in studies analysing T/B infiltrates in kidneys of systemic lupus erythematosus patients^7^,^15^.

To study T/B cooperation in inflamed tissues, we complemented the well-established murine airway inflammation model based on the adoptive transfer of OVA-specific T cells^16^ by an additional transfer of antigen-specific B cells. This novel airway inflammation model allowed us the parallel analysis of antigen-specific T and B cells during early and late phases of the immune response, thus providing new insights into the phenotype and function of T and B cells in peripheral inflamed tissues. The adoptive transfer system enabled us to track antigen-specific T and B cells, and showed that in the chronic phase of the immune response the vast majority of antigen-specific T and B cells were not located in the draining lymph nodes but in the inflamed tissue. Similar to the situation in autoimmune patients, T/B infiltrates in the lung mainly consisted of loose aggregates lacking FDC and any ordered structures. Nevertheless, not only plasma cells but also GC-like B cells were found in these infiltrates. This shows that SLO-like structures and FDC are not absolutely necessary for the development of GC-like B cells. So far, this only has been shown for the very special case of the so called milky spots in the omentum^17^. These structures, important for peritoneal cavity immune responses, completely lack FDC but develop GC B cells on antigen exposure.

Despite the presence of GC-like B cells, no classical TFH cells expressing CXCR5 and Bcl-6 were found in the inflamed lung tissue. Three recent publications demonstrated the presence of CXCR5-positive TFH cells in inflamed lung tissue. However, in one study CXCR5^+^ T cells were directly shown to reside in ectopic lymphoid structures^18^; in the other two studies^19^,^20^ this aspect was not analysed, but both used infection models, where the development of ectopic lymphoid tissue can be presumed. We, however, focused on antigen-specific T cells in loose lymphocytic aggregates, and not in ectopic lymphoid tissue. These lung-infiltrating T cells lacked classical TFH cell markers, but still had potent B-cell helper capacities and were present in close contact with strongly proliferating GC-like B cells. The high frequency of Ki-67-positive B cells indicates that active T/B cooperation takes place. Indeed, a recent detailed clonal tree analysis of B cells located in Ki-67^+^ but FDC-negative T/B aggregates from lupus nephritis patients revealed ongoing somatic hypermutation^7^. These FDC-negative T/B infiltrates in the kidney were first described by our group^15^. Already in this early study we had many indications that active T/B cooperation takes place in the infiltrates and leads to the local generation of plasma cells.

The CXCR5-negative B helper T cells from the lung exhibit some features of other recently described T-cell populations with B helper potential, such as splenic extrafollicular helper T cells found in certain autoimmune mouse models^21^. Despite their non-TFH phenotype with very low CXCR5 expression, these extrafollicular T cells exhibited a strong potential for B-cell help through expression of IL-4, IL-21 and CD40L, and might be important for the extrafollicular plasma cell reaction. The second example is bone marrow-resident CD4^+^ memory T cells that also lack typical TFH features^22^, but are, nevertheless, capable of providing help to B cells for antibody production and affinity maturation. In the rheumatoid joint CXCR5- and Bcl-6-negative T cells were shown to be a source of CXCL13, which might be important for the recruitment of B cells^23^. A very recent publication described CXCR5-negative but IL-21-positive T cells in the inflamed lung^24^.

So why are lung-infiltrating B helper T cells negative for CXCR5? Possibly, because they simply do not need this chemokine receptor to get into contact with B cells. In the unordered infiltrates with interspersed T and B cells as observed in our model, T cells do not have to migrate from a T-cell area into a B-cell follicle as in the lymph node. In addition, expression of the CXCR5 ligand CXCL13 is unlikely in these FDC-negative lung infiltrates, since in the mouse this chemokine is mainly produced by FDC^25^.

Our study did not only identify the lung tissue as a major reservoir for antigen-specific effector cells but also as a niche for long-lived memory B cells. Tissue-resident memory T cells were recently recognized as a discrete memory T-cell subset, which facilitates rapid secondary responses directly at the site of antigen entry^26^. Our study now shows that not only antigen-specific T but also B cells are found in close contact in the lung tissue. So far, only three studies described the presence of memory B cells in the lung^27^,^28^,^29^, and all were viral infection models, which (different to our model) typically include the development of ectopic lymphoid tissue. The persisting T/B infiltrates in the lung might be important in secondary immune responses for the rapid generation of antigen-specific plasma cells directly at the site of antigen entry. Interestingly, antigen-specific memory T and B cells additionally distributed to other classical memory cell niches like spleen and bone marrow. Therefore, immune responses in the lung can give rise not only to local but also systemic protection.

Our detailed analysis of T/B cooperation in inflamed tissues is also highly relevant from a clinical perspective. With improved therapeutic options for autoimmune diseases preventing long phases of chronic disease, the development of ectopic lymphoid tissue as a final stage of tissue infiltration is nowadays only rarely observed. However, FDC-negative, unstructured lymphocytic tissue infiltrates are a frequent pathological finding, and our study shed more light on the interaction of autoreactive T and B cells in these infiltrates.

## Methods

### Mice

OT-II (Jax stock 004194) and Smarta^30^ T-cell receptor transgenic mice or B1-8i mice^31^ (additionally crossed with kappa light chain knockout mice^32^ to ensure NP-specificity of B cells) were crossed to B6PL (Thy-1.1^+^, Jax stock 000406) or Ly-5.1 (CD45.1^+^, Jax stock 002014) mice, respectively. Transgenic strains and wild-type C57BL/6 mice were bred under specific pathogen-free conditions in the animal facility of the Federal Institute for Risk Assessment, Berlin. For experiments female mice in an age between 8 and 16 weeks were used. Animal experiments were conducted according to the German animal protection laws, and approved by the responsible governmental authority (LAGeSo).

### Airway inflammation model

OT-II T cells from spleens were sorted magnetically for CD62L^high^ cells (Miltenyi Biotec), and cultured with splenocytes for 5 days with the addition of OVA peptide, recombinant IL-4, anti-IFN-γ and anti-IL-12. 2 × 10^6^ OT-II T and 2 × 10^6^ B1-8i B cells were adoptively transferred into C57BL/6 mice by intravenous injection, 16–24 h later mice were challenged i.n. with 5 μg NP-OVA conjugate and 5 μg LPS (Sigma) as adjuvant. Challenge with same amounts of antigen was repeated on day 1, 10 and 13. For analysis of memory T cells, naive T cells from Smarta mice were directly co-transferred into recipients. Mice were challenged i.n. with NP and the cognate peptide (P13 GP_61–80_) chemically coupled to mouse serum albumin (20 μg on day 0 and 1, and 5 μg on days 10, 13 and 17) and 5 μg LPS.

### Cell isolation

Lungs were perfused with PBS. Bronchial lymph nodes were dissected from the lung tissue under a microscope and prepared separately for flow cytometric analysis by meshing through a 70-μm sieve. Single-cell suspensions from lung tissue were prepared using the gentle MACS dissociator (Miltenyi Biotec) and digestion with 0.5 mg ml^−1^ collagenase D and 20 μg ml^−1^ DNase I (Roche) for 25 min at 37 °C. Spleens were meshed through a sieve, and bone marrow was flushed out of femur and tibia of hind legs. All cells were counted using a Guava EasyCyte capillary flow cytometer and ViaCount solution (Millipore).

### T/B co-cultures

To compare the B-cell helper qualities of effector T cells from lymph nodes and lung, cells from both organs were enriched with anti-Thy-1.1 microbeads (Miltenyi Biotec) followed by flow-sorting on a FACS ARIA II (BD Biosciences) for CD4^+^ Thy-1.1^+^ Thy-1.2^−^ CD44^high^ CD8^−^ B220^−^ DAPI^−^ cells. Naive T cells from spleens of OT-II mice were sorted as CD4^+^ Thy-1.1^+^ CD62L^+^ B220^−^ DAPI^−^ cells. Naive B cells were sorted from spleens of B1-8i mice as CD19^+^ CD4^−^ DAPI^−^ cells. T and B cells were co-cultured in 96-well round-bottom plates (4–6 individual wells) at a 1:4 ratio in the presence of 10 μg ml^−1^ NP–OVA for 7 days.

### Flow cytometry

Single-cell suspensions from lymph node, lung, spleen or bone marrow were stained with different combinations of fluorophore-conjugated antibodies (Supplementary Table I). Streptavidin-PE-Cy7 (0.25 μg ml^−1^) or -PerCP (0.5 μg ml^−1^) were used as secondary reagents for biotinylated Ab. Fc receptors were blocked with 20 μg ml^−1^ 2.4G2 (anti-CD16/32).

For analysis of cytokine production, cells were re-stimulated *in vitro* with OVA peptide for 4.5 h with Brefeldin A added after 1 h. Following staining of cell surface antigens, cells were fixed with formaldehyde, permeabilized with saponin and stained intracellularly with anti-cytokine antibodies (Supplementary Table I). For detection of IL-21, an IL-21R-Fc chimera, followed by Cy5-conjugated goat anti-human Ig (Jackson ImmunoResearch, 1:800), was used.

For intracellular staining of transcription factors (Supplementary Table I) the FoxP3 Staining Buffer Set from eBioscience was used. For activation-induced cytidine deaminase staining, cells were stained intracellularly before surface staining, and Alexa Fluor 647-conjugated goat anti-rat Ig (Invitrogen, 2.5 μg ml^−1^) was used as secondary reagent. For intracellular staining of Ig, cells were fixed with formaldehyde, permeabilized with saponin buffer and stained with fluorescein isothiocyanate (FITC)-conjugated anti-Ig antibodies (Supplementary Table I).

To discriminate dead cells, either 4,6-diamidino-2-phenylindole (DAPI) was added to live cells immediately before analysis or cells were stained with 1.34 μM Pacific Orange succinimidyl ester (Invitrogen) before fixation. Samples were analysed on an LSR II or LSR Fortessa flow cytometer (BD Biosciences). Analysis gates were set on live cells defined by scatter characteristics and exclusion of doublets and dead cells. In all figures, antigen-specific T cells are defined as DAPI^−^ B220^−^ CD4^+^ Thy-1.1^+^ and B cells as DAPI^−^ CD3/CD8^−^ CD19^+^ CD45.2^−^ CD45.1^+^. Data were analysed with FlowJo Software (Treestar).

### Enzyme-linked immunosorbent assay

NP-specific IgG1 and IgA was measured by enzyme-linked immunosorbent assay. Plates were precoated with NP-coupled BSA, and bound antibodies were detected by using horseradish peroxidase-coupled antibodies to mouse IgG1 or IgA (Southern Biotechnologies) and tetramethylbenzidine as substrate.

### Histology

Lungs were filled with 50% TissueTek OCT compound via the trachea and snap frozen in ice-cold isopentane. The 8-μm cryostat sections of the right lower lobe were cut and fixed with acetone. To reduce unspecific signals, peroxidase inactivation and blocking with Casein Solution (Vector Laboratories) was performed.

For light microscopy, tissue section was stained with FITC-coupled OX-7 (anti-Thy-1.1, 1.25 μg ml^−1^), A20 (anti-CD45.1, 5 μg ml^−1^), 7G6 (anti-CD21/35, 1 μg ml^−1^), unconjugated 281-2 (anti-CD138, 5 μg ml^−1^) or MBP-1 (anti-MBP, kindly provided by J. Lee, Scottsdale, 1:2,500). As secondary reagents, peroxidase-conjugated antisera against FITC, digoxygenin (Roche, 1:300) or rat-IgG (Jackson ImmunoResearch, 1:4,000) were used. Slides were developed using 3-amino-9-ethylcarbazole as substrate, nuclei were counterstained with haematoxyline. A periodic acid–Schiff-staining kit (Carl Roth) was used to stain mucopolysaccharides. Slides were analysed on an Axioskop 2 light microscope equipped with an Axiocam (Carl Zeiss).

For immunofluorescence microscopy, sections were stained with FITC- or Alexa Fluor 546-coupled 7G6 (anti-CD21/35, 1 μg ml^−1^), FITC-coupled A20 (anti-CD45.1, 0.25 μg ml^−1^), digoxygenin-conjugated OX-7 (anti-Thy-1.1, 0.25 μg ml^−1^), digoxygenin-conjugated GL7 (anti-GC B cells, 1 μg ml^−1^) or hamster anti-Ki-67 (Abcam, 1:100), followed by Alexa Fluor 594-coupled anti-hamster Ig (Invitrogen, 1:500). Signals were amplified with peroxidase-conjugated anti-FITC followed by FITC-coupled tyramide (Invitrogen). After inactivation of peroxidase, the second antibody was detected with anti-digoxygenin-peroxidase (Roche, 1:300) and Alexa Fluor 647-coupled tyramide. Nuclei were counterstained with DAPI. Images were captured on an LSM 780 with ZEN2010 imaging software (Carl Zeiss).

### B-cell receptor sequencing

Four FDC-positive and six negative infiltrates (determined by CD21/35 staining on a consecutive section) were isolated from frozen lung sections using an Arcturus Veritas laser capture microdissection system. RNA was isolated using the Arcturus PicoPure RNA isolation kit. The VH186.2 sequence of the transgenic B1-8i B-cell receptor was amplified from cDNA using a universal primer in the signal peptide (5′-GATGGAGCTGTATCATGCTCTTCTTGGCAG-3′) and specific primers for the heavy chain of IgA (5′-CACTGGGTCACTTGACAGAGCTTGTG-3′), IgG1 (5′-GCTGCTCAGAGTGTAGAGGTCAGACTGC-3′) and IgM (5′-TGAAGGAAATGGTGCTGGGCAGG-3′). PCR products were cloned into pJET1.2. Sequences from 144 single clones were analysed for somatic hypermutation.

### Statistical analysis

Experimental groups contained at least five animals (randomly assigned), which routinely provides the statistical power to detect biological significant differences in our experimental system. Data were analysed using GraphPad Prism 5. No data exclusion criteria were used.

## Additional information

**Accession codes:** Unique B cell receptor sequences from FDC-negative and -positive infiltrates have been deposited at the European Nucleotide Archive (www.ebi.ac.uk/ena) under the accession codes LT159851, LT159852, LT159853, LT159854, LT159855, LT159856, LT159857, LT159858, LT159859, LT159860, LT159861, LT159862, LT159863, LT159864 and LT159865.

**How to cite this article:** Vu Van, D. *et al.* Local T/B cooperation in inflamed tissues is supported by T follicular helper-like cells. *Nat. Commun.* 7:10875 doi: 10.1038/ncomms10875 (2016).

## Supplementary Material

Supplementary Information Supplementary Figures 1-7 and Supplementary Table 1
