# Supplementary material for: Local T/B cooperation in inflamed tissues is supported by T follicular helper-like cells
Source: Nat Commun. 2016 Feb 26;7:10875. doi: 10.1038/ncomms10875 (PMC4773445; doi:10.1038/ncomms10875)
Supplement: Supplementary Information — Supplementary Figures 1-7 and Supplementary Table 1 [file ncomms10875-s1.pdf]

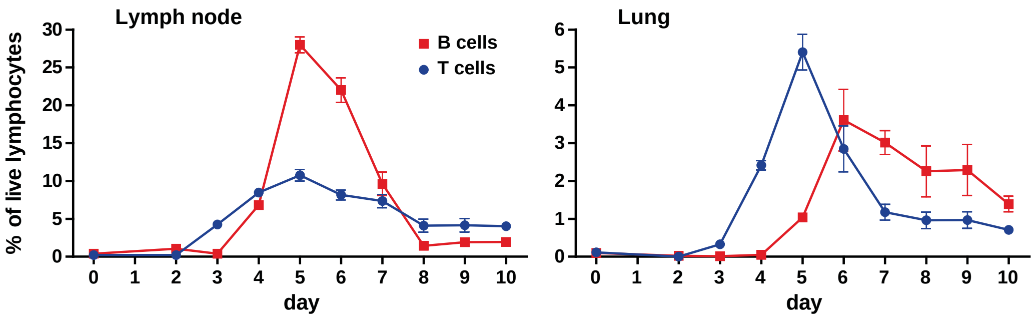

Supplementary Fig. 1: Kinetics of T and B cell expansion in lymph node and lung.
Naive OT-II T cells and B1-8i B cells were adoptively transferred into C57BL/6 recipients, immunized intranasally with NP-OVA on day 0 and 1. Frequencies of transgenic T and B cells in lung-draining lymph node and lung tissue were tracked by flow cytometry. Mean cell frequencies (error bars show SEM) from 3-5 animals per time point and a representative experiment out of two are shown.

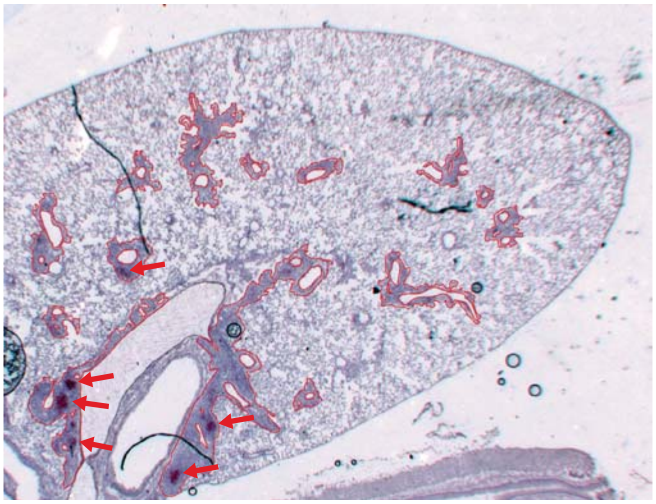

Supplementary Fig. 2: Quantification of FDC-positive areas.
Lungs from two animals were sectioned at different depth and stained for CD21/35 to visualize FDC and hematoxylin to quantify lymphocytic infiltrates. A consecutive section was stained for antigen-specific B cells to prove that all infiltrates contained B cells (data not shown). The total area of infiltrates (red lines) and FDC-positive regions (arrows) was quantified using Zeiss AxioVision software.

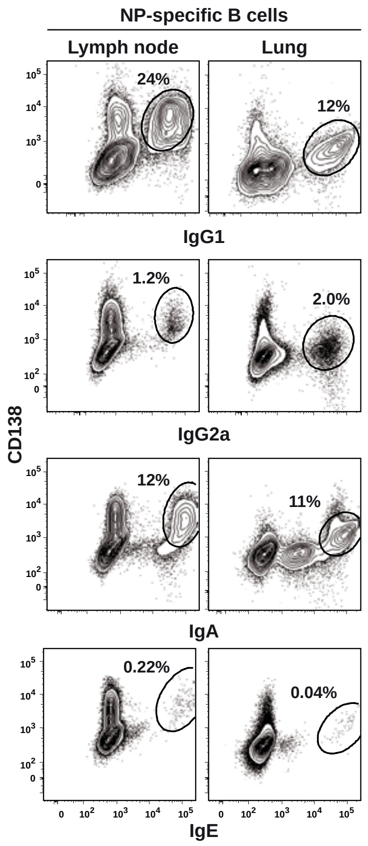

Supplementary Fig. 3: Analysis of immunoglobulin subclasses.
Flow-cytometric analysis (day 17) of antigen-specific B cells from lung-draining lymph nodes and lung tissue. Cells were stained for CD138 and intracellular IgG1, IgG2a, IgA, or IgE. Representative result from two independent experiments with 14 animals.

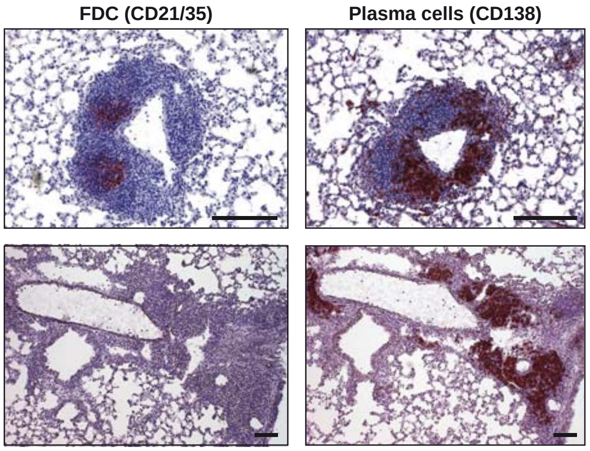

Supplementary Fig. 4: Plasma cells can be found in FDC-positive and negative areas.
Cryosections from inflamed lung-tissue were stained for CD21/35 and CD138. The top row shows serial sections from a FDC-positive and the bottom row from a FDC-negative infiltrate. The bar scales indicate 200 µm.

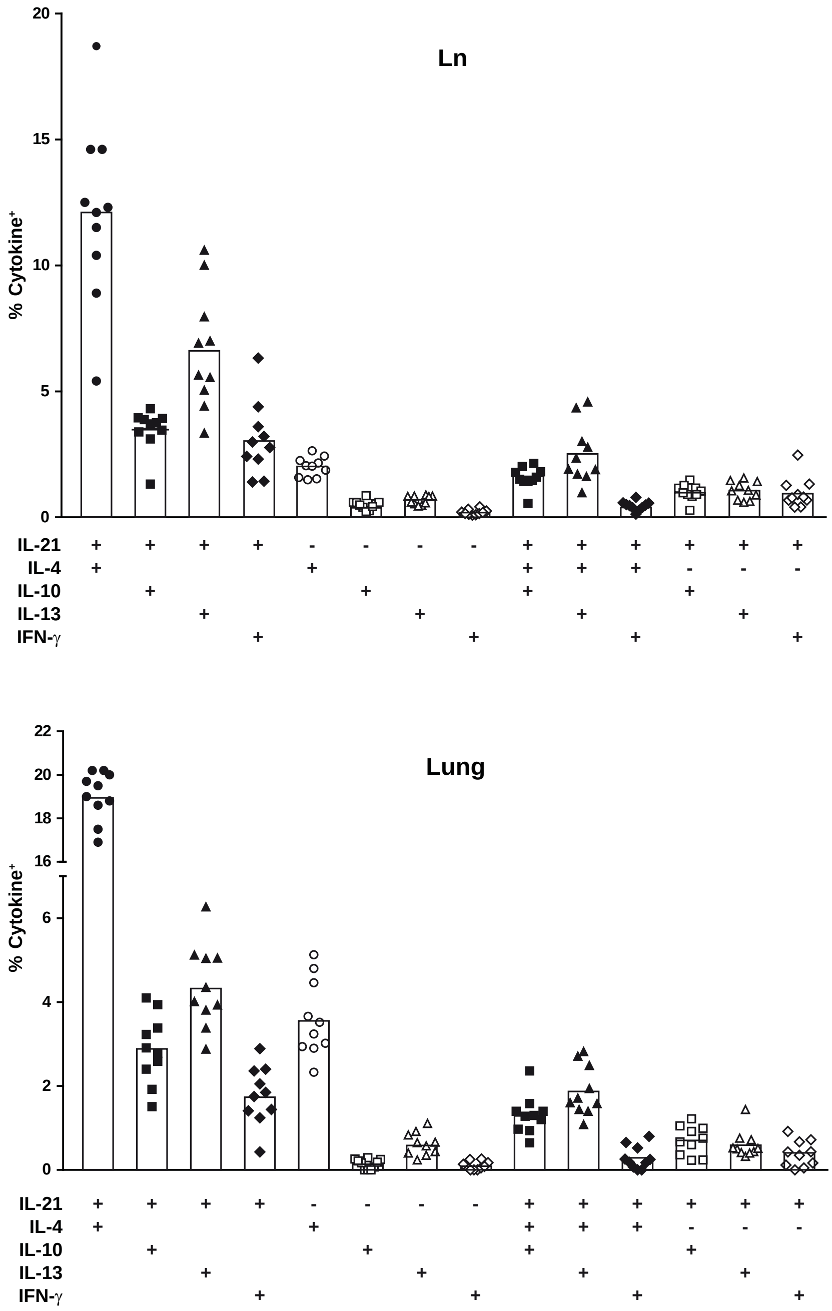

Supplementary Fig. 5: Analysis of cytokine coexpression.

Lung and lung-draining lymph node cells were isolated from a day 17 airway inflammation. Cells were restimulated with OVA-peptide in vitro and antigen-specific T cells were analysed for cytokine production by intracellular staining. Shown is the percentage of T cells expressing the combination of two or three cytokines as indicated. Representative result from 2 independent experiments with 17 animals.

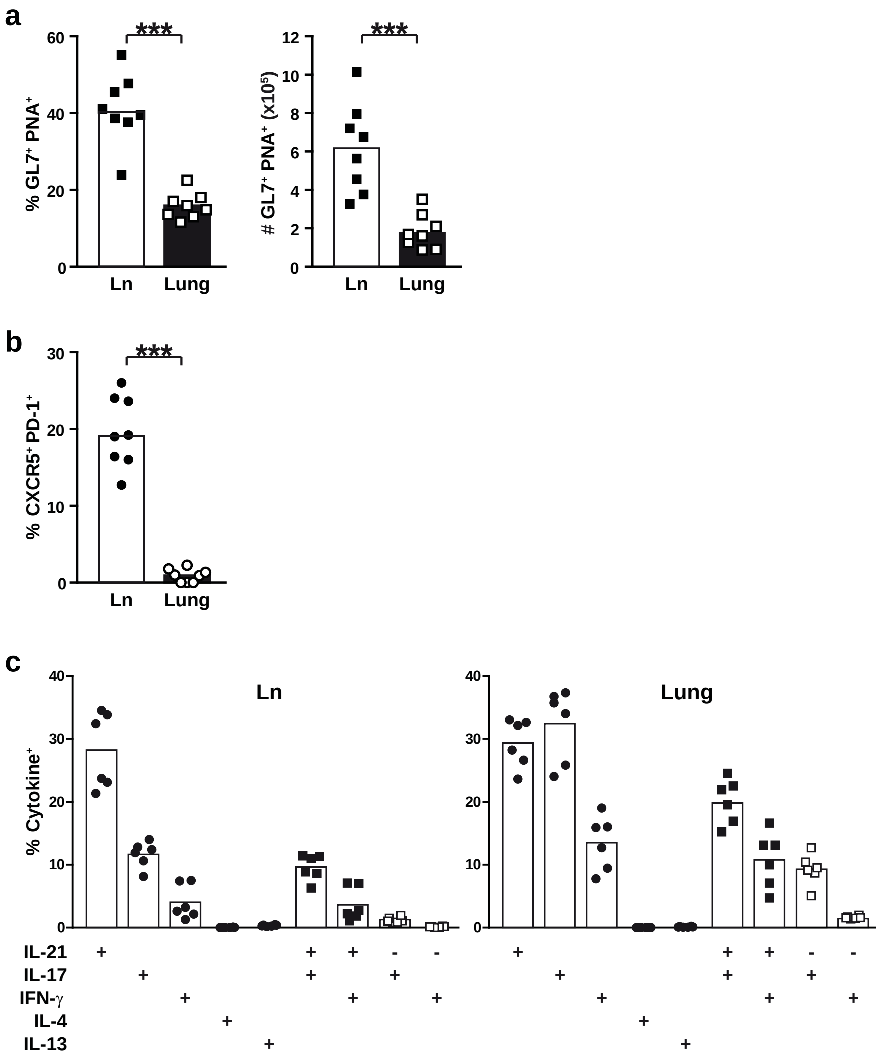

Supplementary Fig. 6: Airway inflammation model with adoptive transfer of naive T cells.
OT-II T cells and B1-8i B cells were co-transferred into C57BL/6 recipients as shown in Fig. 1a except that T cells were not pre-activated in vitro. Mice were challenged with 20 µg NP-OVA + 5 µg LPS on day 0 and 1, and 5 µg NP-OVA + 5 µg LPS on day 10, 13, 16. Lung-draining lymph nodes and lung tissue were analysed on day 20. **(a)** Antigen-specific B cells were analysed for a GC-like phenotype as shown in Fig. 3b. **(b)** Antigen-specific T cells were stained with anti-CXCR5 and PD-1 as shown in Fig. 4a. **(c)** Cells were restimulated with OVA-peptide in vitro and antigen-specific T cells were analysed for cytokine production as shown in Fig. 4c. Shown is the percentage of T cells expressing a single cytokine or the combination of two cytokines. Similar results were obtained using Smarta T cell receptor transgenic T cells instead of OT-II.

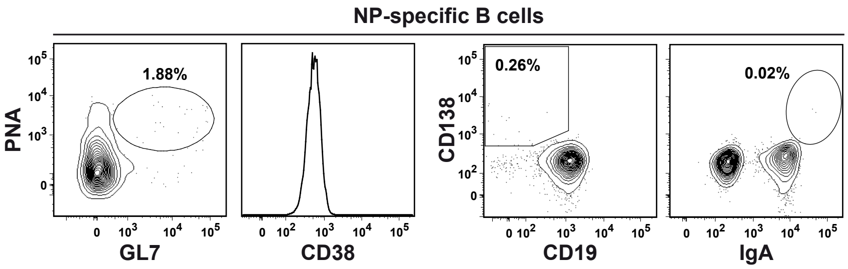

Supplementary Fig. 7: Memory B cells in the lung are no GC or plasma cells.

Flow cytometric characterization of lung-resident antigen-specific B cells at day 41. Representative stainings for the GC B cell marker PNA, GL7, CD38 and the plasma cell marker CD138 in combination with CD19 or IgA on permeabilised cells are shown.

Supplementary Table I: Antibodies used for flow cytometry

Specificity	Clone	Conjugate	Supplier	Concentration
CD3	KT3	Pacific Orange	own conjugate	2 µg/ml
CD4	YTS 191.1	Alexa Fluor 700	own conjugate	0.75 µg/ml
CD4	RM4-5	Brilliant Violet 711	BioLegend	0.25 µg/ml
CD8	53-6.72	APC-eFluor 780	eBioscience	0.5 µg/ml
CD19	6D5	APC-Cy7	BioLegend	0.5 µg/ml
CD19	6D5	Brilliant Violet 785	BioLegend	1:400
CD38	90	Pacific Blue	BioLegend	0.625 µg/ml
CD40L	MR1	PE	BioLegend	2 µg/ml
B220	RA3-6B2	APC-Cy7	BioLegend	0.25 µg/ml
CD45.1	A20	PE-Cy7	BioLegend	0.5 µg/ml
CD45.2	104	Alexa Fluor 700	BioLegend	1.25 µg/ml
CD69	H1.2F3	PerCP	BioLegend	2 µg/ml
CD73	TY/11.8	Brilliant Violet 605	BioLegend	1:200
CD80	16-10A1	Cy5	own conjugate	0.75 µg/ml
Thy-1.1	OX-7	Alexa Fluor 700	own conjugate	0.3 µg/ml
Thy-1.1	OX-7	Pacific Blue	own conjugate	0.2 µg/ml
CD138	281-2	PE	BD Biosciences	0.25 µg/ml
CXCR5	2G8	Biotin	BD Biosciences	5 µg/ml
CCR7	4B12	Biotin	BioLegend	2.5 µg/ml
PD-1	J43	PE	eBioscience	1 µg/ml
PD-L2	TY25	PE	eBioscience	2 µg/ml
ICOS	MIC-2043	Alexa Fluor 647	own conjugate	0.375 µg/ml
IgD	11-26c	Alexa Fluor 647	own conjugate	0.375 µg/ml
IgM	Bet-2	Biotin	own conjugate	2 µg/ml
IgG1	A85-1	PE-CF594	BD Biosciences	0.5 µg/ml
IgG1	LO-MG1-2	FITC	Serotec	0.3 µg/ml
IgG2a	LO-MG2a-9	FITC	Serotec	0.1 µg/ml
IgA	C10-3	FITC	BD Biosciences	0.15 µg/ml
IgE	R35-72	FITC	BD Biosciences	0.625 µg/ml
GC B cells	GL7	Alexa Fluor 647	own conjugate	0.375 µg/ml
GC B cells	PNA	FITC	Vector Laboratories	1.25 µg/ml
IL-4	BVD6-24G2	PE-Cy7	eBioscience	0.125 µg/ml
IL-5	TRFK5	APC	own conjugate	0.25 µg/ml
IL-10	JES5-2A5	PE	own conjugate	1 µg/ml
IL-13	38213.11	Pacific Blue	own conjugate	1.25 µg/ml
IL-17	TC11-18H10	Alexa Fluor 700	BioLegend	0.625 µg/ml
IL-21	IL-21R-Fc	unconjugated	R&D Systems	1 µg/ml
IFN-γ	AN18.17.24	FITC	own conjugate	0.375 µg/ml
Bcl-6	GI191E/A8	PE	eBioscience	0.05 µg/ml
Bcl-6	K112-91	Alexa Fluor 647	BD Biosciences	1:50 µg/ml
GATA-3	TWAJ	PE	eBioscience	0.06 µg/ml
AID	mAID-2	unconjugated	eBioscience	5 µg/ml
